# Southern limits of distribution of the intertidal gobies *Chaenogobius annularis* and *C. gulosus* support the existence of a biogeographic boundary in southern Japan (Teleostei, Perciformes, Gobiidae)

**DOI:** 10.3897/zookeys.725.19952

**Published:** 2017-12-29

**Authors:** Atsunobu Murase, Ryohei Miki, Hiroyuki Motomura

**Affiliations:** 1 Nobeoka Marine Science Station, Field Science Center, University of Miyazaki, 376-6 Akamizu, Nobeoka, Miyazaki 889-0517, Japan; 2 Department of Marine Biology and Environmental Sciences, Faculty of Agriculture, University of Miyazaki, Gakuen-Kibanadai-Nishi, Miyazaki 889-2192, Japan; 3 Interdisciplinary Graduate School of Agriculture and Engineering, University of Miyazaki, 1-1 Gakuen-kibanadai-nishi, Miyazaki 889-2192, Japan; 4 The Kagoshima University Museum, 1-21-30 Korimoto, Kagoshima 890-0065, Japan

**Keywords:** Distributional range, northwestern Pacific, rock pools

## Abstract

Understanding the distributional patterns of individual animal groups with respect to coastal topology and the local physical environment provides essential foundational frameworks for marine zoogeography. In the northwestern Pacific waters of Japan, the distributional pattern of some cool-temperate species of marine fishes suggests the existence of a biogeographic boundary corresponding to a long sandy shore on the eastern coast of Kyushu, southern Japan. The existence of this hypothetical biogeographic boundary was tested by mapping the southern distributional limit of two species of cool-temperate intertidal gobies, *Chaenogobius annularis* and *C. gulosus*, which are endemic to East Asia and common in rock pools within their range in the Japanese Archipelago. Distribution and abundance were assessed by survey of museum collections from south-east Kyushu (i.e., the entire coasts of Kagoshima and Miyazaki prefectures); and a quantitative survey of the abundance of these gobies in rock pools at various sites around the hypothesized boundary on the eastern coast of Kyushu, including the subtropical Tanega-shima Island. The museum collection survey showed different distribution patterns between the two species: *C. annularis* was distributed along the entire coasts of south-east Kyushu including subtropical islands, whereas *C. gulosus* was distributed along these coasts, including one site on a subtropical island, except for an area south of the hypothesized boundary on the eastern coast of Kyushu. The density and occurrence rates of *C. annularis* in rock pools decreased with latitude, it being absent from a subtropical island, and *C. gulosus* was not detected from sites south of the hypothesized boundary. The qualitative survey showed that the southernmost records of *C. annularis* and *C. gulosus* were the adjacent subtropical islands (Yaku-shima and Tanega-shima islands respectively), although the quantitative survey suggested that their normal range of distribution was limited to the southern part of the Kyushu mainland. A combination of qualitative and quantitative survey methods in the present study highlighted that the southernmost record of a certain species may not necessarily indicate the true limit of its distribution. The distribution of *C. gulosus* supports the existence of the hypothetical biogeographic boundary, and the different distribution patterns of the two species may be caused by differences in their early life histories.

## Introduction

The delineation and characterization of the marine fauna is one of the objectives of marine zoogeography (Briggs 1974) and understanding the distribution patterns of individual animal groups is essential for this work. When a species’ distribution is considered on a geographical, habitat, or microhabitat scale, its limits may be surrounded by areas where the species cannot maintain a population due to adverse physical conditions or a lack of resources permitting survival (Cox and Moore 2005). Indeed, a strong link between species distribution and climatic areas is universally observed in the coastal zone (Briggs 1974, 1995, Nishimura 1992, Cox and Moore 2005, Briggs and Bowen 2012). According to these biogeographic foundations, the distribution of a certain species can be used to construct baseline information for delineating any biogeographic boundaries that correspond to topographic and/or climatic features. Accurate range limit data can reveal range boundary disequilibrium, where a species’ distribution and its potential geographic distribution differ. Such discrepancies may reflect differences in dispersal, life history, recolonization history, or stochasticity (Sexton et al. 2009).

Intertidal organisms are generally easily accessible, and readily available to be sampled and used as ecological and biogeographic indicators (Raffaelli and Hawkins 1996). Biogeographic analyses using intertidal macrobenthic fauna have detected faunal similarities and relationships relating to coastal environments in the north-western Pacific region (Asakura and Suzuki 1987, Nakaoka et al. 2006, Kurihara 2007, Ohgaki 2011). Intertidal fishes are also likely to be useful indicator species for coastal biogeography in terms of their taxonomic arrangement and distribution information, which can lead to a biogeographic categorization for each species (Murase 2013, Okada et al. 2015).

**Figure 1.**
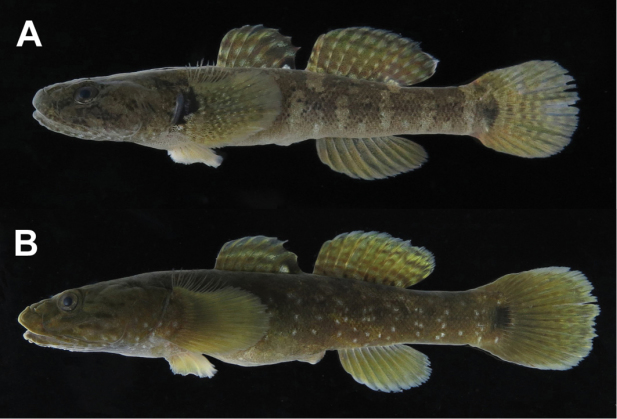
Fresh specimens of the two species of *Chaenogobius*: **A** *Chaenogobius annularis*, KPM-NI 42850 (photo number, KPM-NR 179153), 52.7 mm SL, Nobeoka City, Miyazaki Prefecture **B** *Chaenogobius gulosus*, KPM-NI 42951 (KPM-NR 179221), 73.0 mm SL, Kadogawa Bay, Miyazaki Prefecture. Photos by A. Murase.

**Figure 2.**
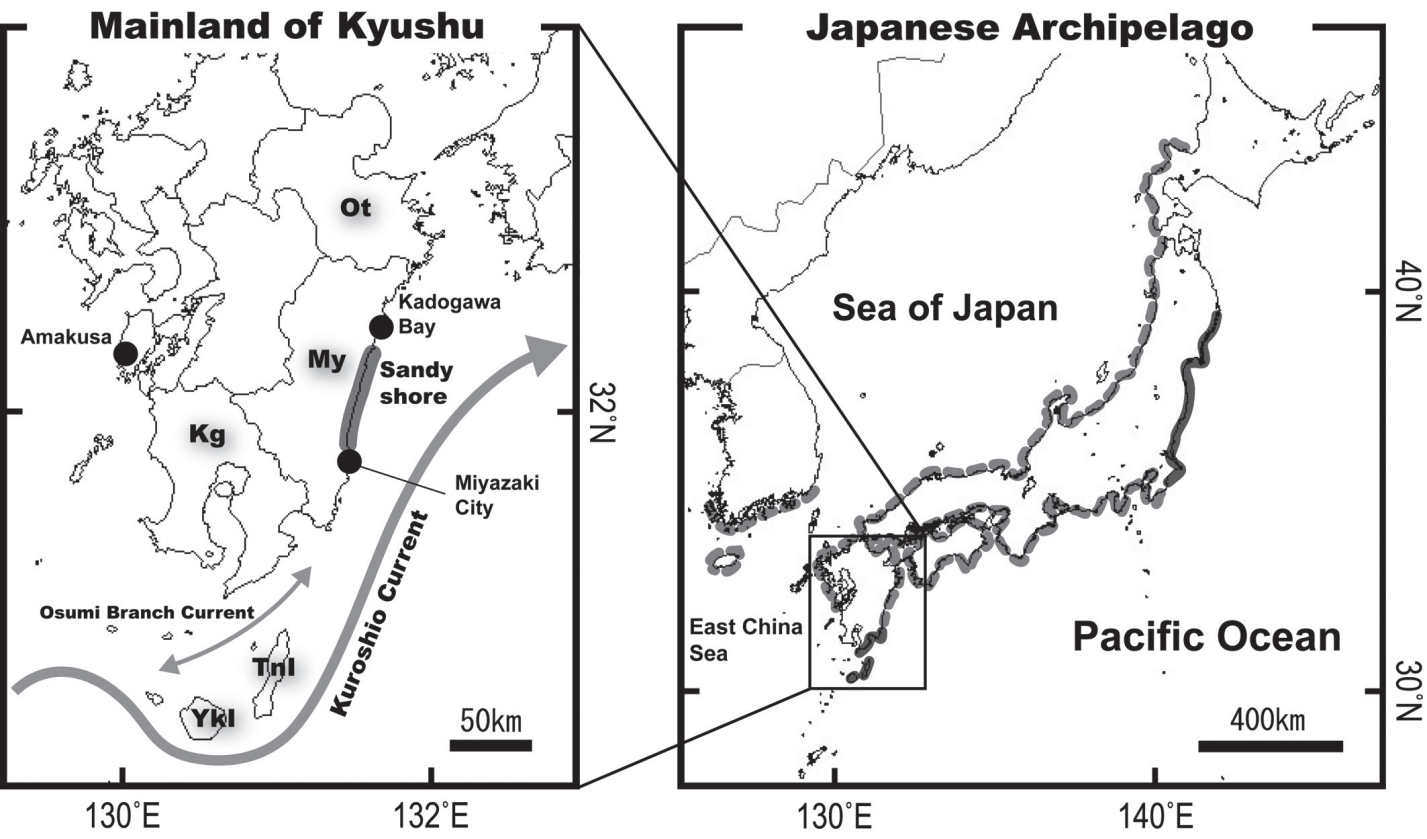
Conventional information on the distributional range of the two species of *Chaenogobius* (sensu Akihito et al. 2013 and Kwun et al. 2017) and a map of mainland Kyushu, southern Japan, showing the position of a long sandy shore on the eastern coast of Kyushu (a bold gray line on left-hand map). The grey-dash and solid lines on the Japanese Archipelago with adjacent areas (right-hand map) indicate the distributional range shared by the two species and that of only *C. annularis* respectively. Abbreviations for prefecture and island names mentioned in the text are as follows: **Ot** Oita Prefecture **My** Miyazaki Prefecture **Kg** Kagoshima Prefecture **TnI** Tanega-shima Island **YkI** Yaku-shima Island.

The East Asian endemic genus *Chaenogobius* Gill, 1859, is composed of two species, *C. annularis* Gill, 1859, and *C. gulosus* (Sauvage, 1882) (Fig. 1), which are distributed along the temperate coasts of Japan and the Korean Peninsula (Stevenson 2002, Akihito et al. 2013, Kwun et al. 2017). Both species inhabit intertidal rocky shores and rock pools, and are common and/or the predominant species throughout the temperate region of the Japanese Archipelago (Sasaki and Hattori 1969, Nakamura 1970, Okamura and Amaoka 1997, Arakaki and Tokeshi 2006, Murase et al. 2010; Amaoka et al. 2011, Kohno 2011). Nakabo (2013), in assigning each Japanese fish species to its biogeographic affinity, classified the genus *Chaenogobius* as a category of “shallow rocky-reef fishes in the continental coast of cool-temperate water area (thereafter represented as “cool-temperate species” in this text)”. This group occurs in the following areas: Tsugaru strait southward to southern Kyushu along the Sea of Japan coast, Aomori Prefecture to Miyazaki Prefecture along the Pacific coast, and the southern Korean Peninsula. In addition, according to fig. 2B of Nakabo (2013), the northern part of Miyazaki Prefecture is the southern distributional limit of this group on the Pacific coast. *Ditrema temminckii* Bleeker, 1853 (Embiotocidae), a species within this biogeographic group, has been reported from Kadogawa Bay in the northern part of Miyazaki Prefecture, representing its southern limit on the Pacific coast (Murase et al. 2017). Nakabo’s results, and the known distribution of other members of cool-temperate species, suggest the existence of a biogeographic boundary between the northern and southern parts of the eastern coast of Kyushu. This hypothetical biogeographic boundary casts doubt on the previous understanding of the distribution of the two species of *Chaenogobius*. Akihito et al. (2013) summarized the species’ distribution as the temperate region of the Japanese Archipelago including almost the entire coast of Kyushu: *C. annularis* ranging from Otaru, western Hokkaido, southward to Amakusa, Kumamoto Prefecture, along the Sea of Japan/East China Sea coast, Otsuchi, Iwate Prefecture to Yaku-shima Island along the Pacific coast, and the southern Korean Peninsula; and *C. gulosus* ranging from Yoichi Town, western Hokkaido, southward to Amakusa, Kumamoto Prefecture along the Sea of Japan/East China Sea coast, Kominato, Chiba Prefecture to Miyazaki City, Miyazaki Prefecture along the Pacific coast, and the southern Korean Peninsula (Fig. 2). The two species are common in Japanese waters and are likely to be important for biogeographic analyses, not only in comprehensive ichthyofaunal surveys but also as components of intertidal fish assemblages. In order to clarify the southernmost limit of distribution of the two species of *Chaenogobius* with regard to the abovementioned hypothetical biogeographic boundary on the eastern coast of Kyushu, the present study re-examines the distribution of these species in south-east Kyushu (Kagoshima and Miyazaki prefectures including subtropical adjacent islands (Fig. 2) on the basis of voucher specimens deposited in public museums. To further test the existence of this biogeographic boundary for cool-temperate species, we analysed also the results of rock pool surveys to estimate the occurrence and abundance of these gobies at various sites along the eastern coast of Kyushu.

## Materials and methods

### Qualitative survey

Laboratories of the Kagoshima University Museum and the Nobeoka Marine Science Station, University of Miyazaki, have collected specimens of fish species inhabiting coastal zones of Kagoshima and Miyazaki prefectures, south-east Kyushu. The specimens were deposited in fish collections of public museums, both the Kagoshima University Museum (KAUM–I.) and the Kanagawa Prefectural Museum of Natural History (KPM-NI). Curatorial procedures for those specimens followed Motomura and Ishikawa (2013). Specimens of the genus *Chaenogobius* were examined in the Kagoshima University Museum (KAUM–I.) and the Kanagawa Prefectural Museum of Natural History (KPM-NI), and re-identified following Akihito et al. (2013) and Harada (2014). Standard length (SL: distance from tip of snout to posterior edge of hypural plate) was measured on each specimen. Collecting localities were mapped as distribution records. Specimens examined are listed in Suppl. materials 1 and 2.

### Study sites for quantitative survey

**Figure 3.**
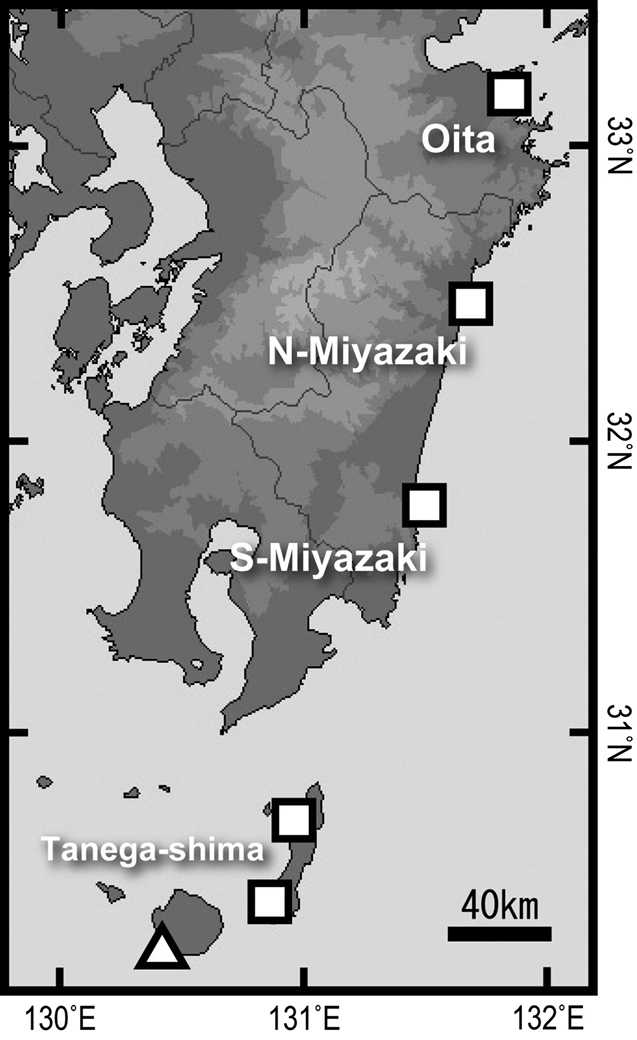
Map showing the sampling sites in the present study (squares) and the site of a previous study (triangle, Yaku-shima Island: Murase 2013, 2015) for the quantitative survey in rock pools along the eastern coast of Kyushu, southern Japan.

A long, sandy shore, measuring about 50 km in length from north to south, lies at the centre of the Miyazaki Prefecture coast (Fig. 2). The abovementioned southernmost limit of the cool-temperate fish species on the Pacific coast side of Kyushu is just north of the upper part of this sandy shore. We herein define the position of this sandy shore as a hypothetical biogeographic boundary (HBB) for members of the cool-temperate fish species. In each of the northern and southern regions of the HBB, two rocky shore sampling sites were selected (total 4 sites), including the Osumi Islands, to examine the occurrence of the two species of *Chaenogobius* near the HBB (Fig. 3). Each site was separated by roughly the same distance (ca. 50–70 km). The sites were as follows: the northernmost site (33°09'53"N, 131°49'27"E) was in Shitanoe, Usuki City, Oita Prefecture (hereafter Oita) at the middle of the western coast of the Bungo Strait, northern part of eastern coast of Kyushu; the second site, on the Pacific coast of Kyushu (32°28'16"N, 131°40'58"E), was Kaneiso Beach, northern coast of Kadogawa Bay, Miyazaki Prefecture (hereafter N-Miyazaki), north of the HBB; the third site (31°47'40"N, 131°28'37"E) was Oryuzako, Miyazaki City, Miyazaki Prefecture (hereafter S-Miyazaki), located at the southern part of Pacific coast of Kyushu, south of the HBB; the southernmost sites (30°41'02"N, 130°57'10"E and 30°25'12"N, 130°51'49"E) were both on Tanega-shima Island (one of the Osumi Islands, hereafter Tanega-shima), located ca. 20 km southeast of the tip of Osumi Peninsula, Kagoshima Prefecture. Data from the two Tanega-shima sites were combined as one site. All the sites were selected to be as uniform as possible with respect to the physical environment (such as wave exposure).

### Methods for quantitative survey

Generally, five rock pools were sampled at each designated sampling site, except for the two sites on Tanega-shima Island, where 2–4 rock pools were sampled at each site. The total number of rock pools investigated during the seasons differed at each site, as the same pools were not sampled each time; some tide pools were inaccessible on certain sampling occasions due to costal weather conditions, such as high waves, and at these times, a neighbouring rock pool was selected. Across the sampling sites, tide pools were selected to be as similar as possible in mean size and elevation, following the methods of Castellanos-Galindo et al. (2005) for rock pool measurements and volume estimations. Rock pool samplings were conducted near the spring tide in Japanese spring (late March–May) and autumn (late September–October) 2016 at each site. These seasons were chosen because juveniles of *Chaenogobius* are abundant in spring on shallow rocky shores, and these gobies appear to settle by autumn (Harada 2014; Murase, pers. obs.). Therefore, these are the best seasons for comparing goby abundance between the sites in terms of both juvenile and mature individuals. Fish sampling was performed once at each rock pool in each season by completely emptying the rock pool with a bucket and a mug as in Murase (2013). Just after collection, the fishes were immediately placed in a plastic bag with ice and sea water, and transported to the laboratory. The specimens were preserved in 70 % ethanol after fixation with 10 % formalin. Specimens were identified according to Akihito et al. (2013) and Harada (2014), and measured (SL). All specimens were deposited in KPM-NI as vouchers for the quantitative survey, and listed in Suppl. materials 3 and 4. Density (individuals per m^3^) of each species at each rock pool was calculated using the number of individuals and the estimated volume of each rock pool. To ascertain the variation in fish density between the sites, one-way analysis of variance (ANOVA) and a Tukey’s test or t-test were performed for each season after data were log_10_ (x + 1.0) transformed. Adding to this, the occurrence rates of the two species at each site for each season were calculated as the “frequency of occurrence in the species within a site (number of rock pools where the species was present)/frequency of sampling event within the site (total number of rock pools investigated at the site) × 100 (%)”.

## Results

**Figure 4.**
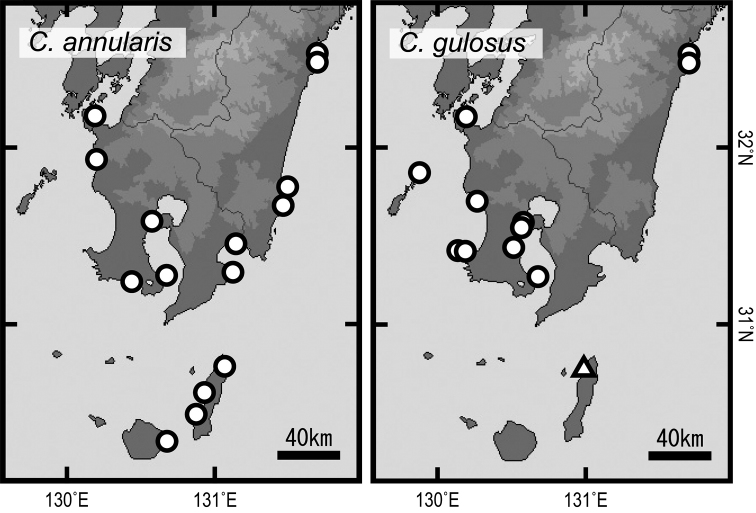
Records (circles and a triangle) of *Chaenogobius annularis* (left) and *C. gulosus* (right) in south-east Kyushu, southern Japan, based on the examination of museum specimens (qualitative survey). A triangle indicating occurrence of *C. gulosus* in Tanega-shima Island by possible human-induced transportation.

Geographical distribution obtained from museum specimens is shown in Fig. 4. *Chaenogobius annularis* is distributed along almost the entire coasts of Kagoshima and Miyazaki prefectures, south-east Kyushu including two of the Osumi Islands (Yaku-shima and Tanega-shima islands). Museum data also resulted in new records for the species on the East China Sea coasts, southern part of Satsuma Peninsula and Kinko Bay, Kagoshima Prefecture. The distribution of *C. gulosus* was also extended, from the coasts of the East China Sea to Kinko Bay in Kagoshima prefecture, but on the eastern coast of Kyushu it was restricted to the northern part of Miyazaki Prefecture (Kadogawa Bay and Nobeoka City), in the northern area of the HBB (Fig. 2). Two adult individuals of *C. gulosus* were recorded from the northwestern coast of Tanega-shima Island (Fig. 4, Suppl. material 2), representing the southernmost record of the species. We re-identified the specimen of *C. gulosus* reported from Miyaziki City by Akihito et al. (2013) (KAUM–I. 21427, Masahiro Aizawa personal communication) as *C. annularis*.

**Figure 5.**
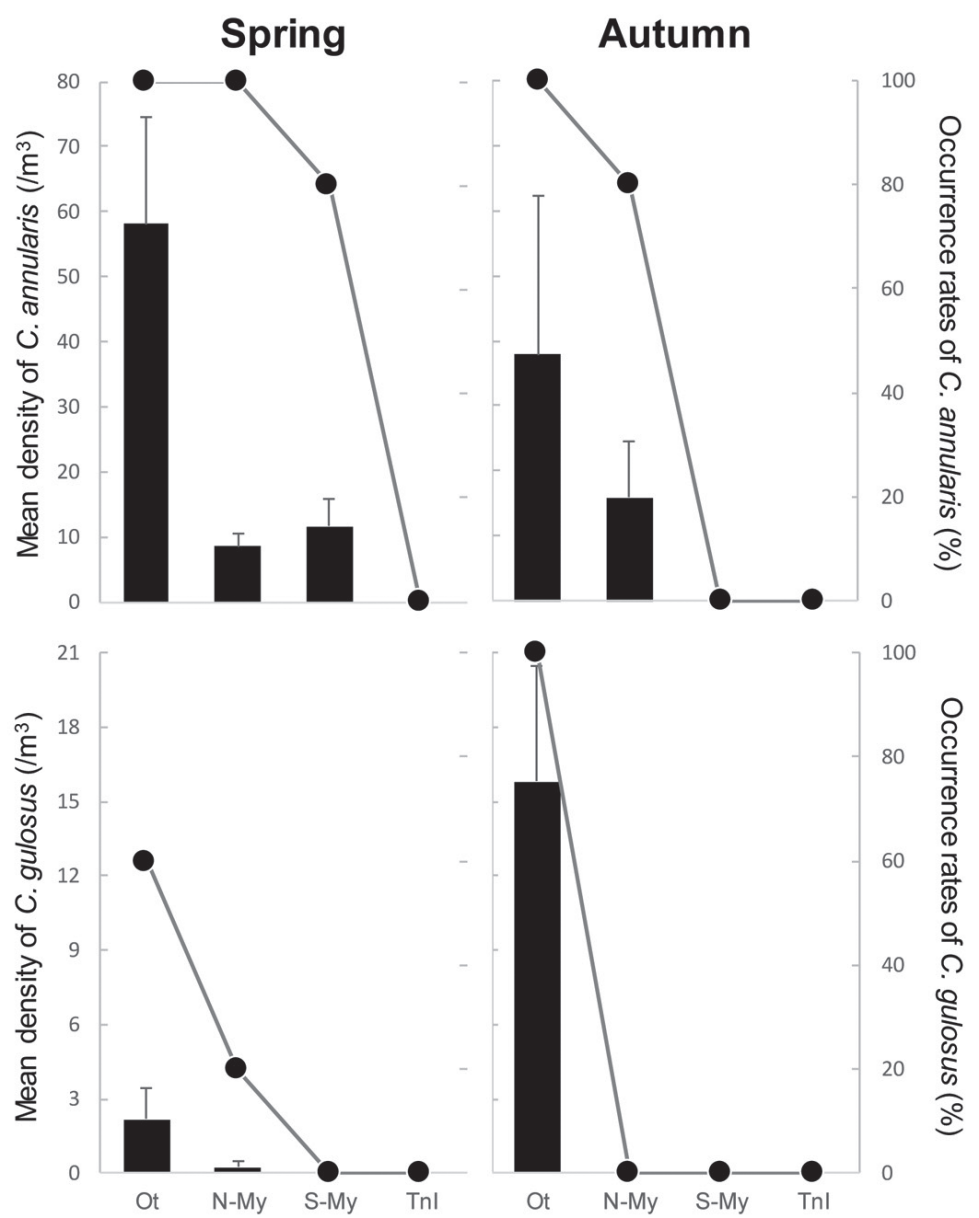
Results of quantitative samplings in rockpools at four sites on the eastern coast of Kyushu, southern Japan. Solid bars and plots show mean density (error bars indicating standard error) and occurrence rates of each species of *Chaenogobius* (upper, *C. annularis*; lower, *C. gulosus*) at each site in the two seasons (left, spring; right, autumn) respectively. Abbreviations of the sites on the x-axis are as follows: Ot, Oita; N-My, N-Miyazaki, S-My, S-Miyazaki; TnI, Tanega-shima (these locality names correspond to those used in Fig. 3). Sample sizes, *n* = 5, except for Tanega-shima in autumn (*n* = 8).

Rock pool volumes for the quantitative survey ranged from 0.06 to 0.76 m^3^ (*n* = 5, mean ± SD: 0.47 ± 0.33 m^3^), 0.31 to 1.01 m^3^ (*n* = 5, 0.72 ± 0.32 m^3^), 0.08 to 5.92 m^3^ (*n* = 7, 1.73 ± 2.54 m^3^), and 0.12 to 4.13 m^3^ (*n* = 8, 1.14 ± 1.33 m^3^) in Oita, N-Miyazaki, S-Miyazaki, and Tanega-shima respectively. Rock pool heights (pool surface height at low tide; see fig. 1 in Murase et al. 2010) ranged from 0.52 to 1.24 m (*n* = 5, mean ± SD = 0.79 ± 0.30 m), 0.60 to 1.41 m (*n* = 5, 0.95 ± 0.35 m), 0.69 to 1.08 m (*n* = 7, 0.90 ± 0.14 m), and 0.76 to 1.66 m (*n* = 8, 1.12 ± 0.28 m) in Oita, N-Miyazaki, S-Miyazaki, and Tanega-shima respectively. The quantitative survey revealed variation and a gradient of occurrence pattern in the two species of *Chaenogobius* along the eastern coast of Kyushu (Fig. 5). There were no individuals for each species in the following sites and seasons: *C. annularis* at S-Miyazaki in autumn and at Tanega-shima sites in any season; *C. gulosus* at N-Miyazaki in autumn, at S-Miyazaki and Tanega-shima sites in any season. Based on this, the values of non-individual sites were omitted from the descriptions of size range and statistical analyses. The density of *C. annularis* decreased from Oita to Tanega-shima in each season. In spring, the density at Oita (58.0 ± 16.4 per m^3^ = average ± SE) was significantly higher than that at N-Miyazaki (8.5 ± 1.9 per m^3^) and S-Miyazaki (11.6 ± 4.2 per m^3^) (Tukey’s test: *p* < 0.05 in each comparison). In autumn, the density at Oita (37.9 ± 24.4 per m^3^) tended to be higher than that at N-Miyazaki (15.9 ± 8.6 per m^3^) but no significant difference was detected (t-test: *p* = 0.3663). The occurrence rates of *C. annularis* increased with latitude from zero to 100 %, reflecting the density pattern in each season (Fig. 5). The density of *C. gulosus* did not significantly differ between Oita and N-Miyazaki in the spring (t-test: *p* = 0.1375) but only a single individual was collected at a single rock pool in N-Miyazaki by the quantitative method. Correspondingly, the occurrence rates of *C. gulosus* were higher in Oita (60 and 100 % in spring and autumn respectively) than in N-Miyazaki (20 % in spring).

## Discussion

**Figure 6.**
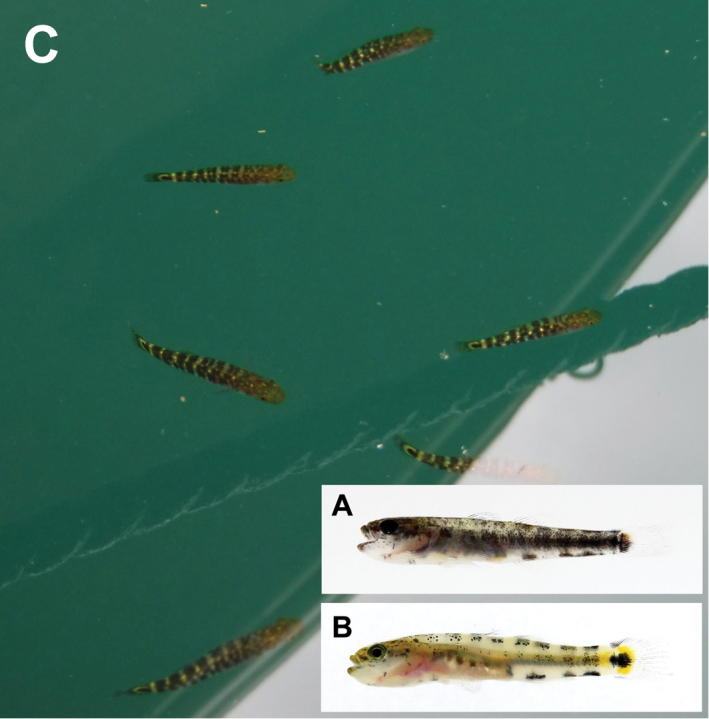
Images of juveniles of the two species of *Chaenogobius* on the coasts of Kyushu, southern Japan: **A** *Chaenogobius annularis*, fresh specimen, KPM-NI 42964 (photo number KPM-NR 179226), 17.6 mm SL, collected at rockpool environment in Usuki City, Oita Prefecture **B** *Chaenogobius gulosus*, fresh specimen, un-catalogued specimen (personal collection number, UMNB-I 3941), 19.2 mm SL, collected with seine net at a small beach in Totoro Port, Nobeoka City, Miyazaki Prefecture, April 2017 **C** *Chaenogobius gulosus*, swimming individuals in Tomioka Port, Reihoku Town, Amakusa, Kumamoto Prefecture, May 2017. Photos by A. Murase.

The qualitative and quantitative surveys showed different distribution patterns between the two species of *Chaenogobius*. The qualitative survey showed *C. annularis* to be distributed along almost the entire coasts of south-east Kyushu including Osumi Islands (both Yaku-shima and Tanega-shima islands), whereas the distribution of *C. gulosus* on the eastern coast of Kyushu was restricted to a northern area around Kadogawa Bay, north of the HBB, and among Osumi Islands; this species was recorded from only a single site at Tanega-shima Island (Fig. 4). The quantitative surveys supported these distribution patterns, with *C. annularis* detected from the three sites of the eastern coast of Kyushu, but *C. gulosus* was detected only from north of the HBB on the eastern coast of Kyushu (Fig. 5). This distribution pattern of *C. gulosus* corresponds well with that of the cool-temperate species in Nakabo’s (2013) fig. 2B and suggests a range boundary disequilibrium (sensu Sexton et al. 2009) on the coast of Kyushu: the species is present on the western side of Kyushu while being absent from the eastern side even at the same latitude. The coastal environment of Miyazaki Prefecture is characterized by two main features, which the western coast of Kyushu does not have: a long sandy shore, oriented north to south, located in the mid-prefecture; and located at the upper reaches of the Kuroshio Current in a domestic temperate region (Fig. 2). Furthermore, the average surface temperature in 2016 was significantly lower in Kadogawa Bay than in Miyazaki City (*n* = 51, paired t-test, *p* < 0.0001: Hydrographic and Oceanographic Department 2017). The long sandy shore provides a non-reef environment where reef fishes cannot settle and inhabit, and the warm strong Kuroshio Current may construct the latitudinal temperature gradient along the coasts of Miyazaki Prefecture. This combination of environmental factors can be a biogeographic barrier for cool-temperate fish species to move from north to south. On the other hand, no such barrier effect on *C. annularis* was detected in terms of its distributional pattern. Observed differences in the distributional pattern of the two species may be caused by their early life history. *C. annularis* occurred in rock pools as juvenile stages prior to settlement (10–20 mm in body length), whereas *C. gulosus* occurred in rock pools as larger individuals after settlement (> 20 mm in body length) (Fig. 6, Suppl. materials 1–4, Sasaki and Hattori 1969). Juveniles of *C. gulosus* have generally been observed out of rock pools, such as in inner zones of fishing ports and near beaches of inner small bays of both eastern and western coasts of Kyushu (Murase unpublished data; Fig. 6B, C). The rock pool environment is physically regulated by the tidal cycle and is unstable depending on its vertical position and size (Metaxas and Scheibling 1993, Raffaelli and Hawkins 1996). These facts might mean that juveniles of *C. gulosus* have a lower tolerance for intertidal environments than *C. annularis*, especially with respect to variation of water temperature. If so, the multiplier effects of a long sandy shore and a high-temperature current may explain the range boundary disequilibrium for *C. gulosus* on the coasts of Kyushu. In addition to this difference among the species of *Chaenogobius*, records of *C. annularis* decreased from Oita to Tanega-shima, and no individuals were detected at S-Miyazaki in autumn during the quantitative survey (Fig. 5); this might indicate an inverse negative relationship with latitudinal environmental gradients for this species. Detailed information on the different physical tolerances of the two species, and the effects of the physical environment on them, is needed for understanding the relationships between these intertidal gobies and the coastal environment.

Senou et al. (2006) were the first to compare the composition of coastal fish faunas along the Kuroshio Current region of Japan by analysing data from 12 sites. Their results revealed two clusters: the Ryukyu Islands and the temperate mainland of Japan including the Ogasawara Islands. The fact that the fish fauna of Tanega-shima Island shares some temperate species with mainland Japan indicates that Tanega-shima Island is clustered with temperate mainland Japan whereas Yaku-shima Island, one of the Osumi Islands, does not share those temperate species and should be clustered with the tropical Ryukyu Islands (Motomura et al. 2010, Motomura 2015, Motomura and Harazaki 2017). The qualitative survey of the present study supports these clusters on the basis of the presence of *C. gulosus* in Tanega-shima and the absence of the species from Yaku-shima Island, although *C. annularis* was present at both islands. However, the present study compared the abundance of certain coastal-fish species on the mainland of Kyushu with the subtropical Osumi Islands located southward from the mainland, and detected no species of *Chaenogobius* at Tanega-shima. Furthermore, a year-round quantitative survey on Yaku-shima Island did not record any species of *Chaenogobius* (Murase 2013, 2015) although individuals of the genus were recorded at all three sites on mainland Kyushu (Fig. 5) in the present study. These facts indicate that the occurrence of species of *Chaenogobius* in the Osumi Islands is occasional and that these subtropical islands are a non-preferred region for these cool-temperate species. The reason for their occurrence at the Osumi Islands could be physical or human-induced transportation. Because *C. annularis* is distributed on the coasts of the Osumi Peninsula, the southernmost part of Kyushu, this species can be transported from the southern part of Kyushu to the Osumi Islands by the Osumi Branch Current, which flows irregularly and bi-directionally between those islands and the southernmost area of Kagoshima Prefecture (Fig. 2; Motomura et al. 2010). However, the occurrence of *C. gulosus* in Tanega-shima does not correspond to the above biogeographic interpretation regarding species distribution and the currents around the southern coast of Kyushu because *C. gulosus* is not distributed in any area associated with the Osumi Branch Current (i.e., the coasts of the Osumi Peninsula). Some Japanese gobiid fish species are known to have been transported overseas via ship ballast water (Okiyama, 1985), and numerous large ferries ply the waters between Kagoshima City and Nishino-omote City on the northwestern part of Tanega-shima Island every week (e.g., Ferry Hibiscus, http://www.yakushimaferry.com/). Because *C. gulosus* is usually present in port areas as juveniles (Fig. 6C), and is distributed on the coasts of Kagoshima City, ballast water may be a more reasonable explanation for its occurrence at Tanega-shima. A combination of qualitative and quantitative survey methods in the present study highlighted that the southernmost record of a certain species may not necessarily mean the true limit of its distributional range.

The distribution patterns and proportion of occurrence in the two species of *Chaenogobius* reported in this study indicate the difference of distribution range of the two species at their southernmost limit, even though they are both categorized as cool-temperate species, and provided implications for the different effects of a biogeographic barrier to each species. In addition to a species/genus level test, comprehensive ichthyofaunal surveys along the coast of Kyushu using qualitative and quantitative methods could clarify the effects of the biogeographic barrier suggested here, and as well as further define variation in physical factors along the Kuroshio Current that influence the formation of coastal fish communities.

## Supplementary materials

Supplementary material 1 List of voucher specimens of *Chaenogobius annularis* for distribution records in south-east Kyushu by examination of museum collections Data type: PDF Explanation note: Lots of specimens ordered according to longitude. Number of KPM-NR indicates photographs of fresh specimens deposited in KPM. This is an open access article distributed under the terms of the Creative Commons Attribution License (CC BY 4.0), which permits unrestricted use, distribution, and reproduction in any medium, provided the original author and source are credited.Atsunobu Murase, Ryohei Miki, Hiroyuki Motomura

Supplementary material 2 List of voucher specimens of *Chaenogobius gulosus* for distribution records in south-east Kyushu by examination of museum collection Data type: PDF Explanation note: Lots of specimen ordered according to longitude. Number of KPM-NR indicating photographs of fresh specimen deposited in KPM. This is an open access article distributed under the terms of the Creative Commons Attribution License (CC BY 4.0), which permits unrestricted use, distribution, and reproduction in any medium, provided the original author and source are credited.Atsunobu Murase, Ryohei Miki, Hiroyuki Motomura

Supplementary material 3 List of voucher specimens of *Chaenogobius annularis* from quantitative surveys at rockpools on the eastern coasts of Kyushu Data type: PDF Explanation note: KPM-NI and KPM-NR indicates the number of the specimen and its photograph respectively. This is an open access article distributed under the terms of the Creative Commons Attribution License (CC BY 4.0), which permits unrestricted use, distribution, and reproduction in any medium, provided the original author and source are credited.Atsunobu Murase, Ryohei Miki, Hiroyuki Motomura

Supplementary material 4 List of voucher specimens of *Chaenogobius gulosus* from quantitative surveys at rockpools on the eastern coasts of Kyushu Data type: PDF Explanation note: KPM-NI and KPM-NR indicates the number of the specimen and its photograph respectively. This is an open access article distributed under the terms of the Creative Commons Attribution License (CC BY 4.0), which permits unrestricted use, distribution, and reproduction in any medium, provided the original author and source are credited.Atsunobu Murase, Ryohei Miki, Hiroyuki Motomura
