# Supplementary material for: Southern limits of distribution of the intertidal gobies Chaenogobius annularis and C. gulosus support the existence of a biogeographic boundary in southern Japan (Teleostei, Perciformes, Gobiidae)
Source: Zookeys. 2017 Dec 29;(725):79–95. doi: 10.3897/zookeys.725.19952 (PMC5769740; doi:10.3897/zookeys.725.19952)
Supplement: Supplementary material 1 — List of voucher specimens of Chaenogobius annularis for distribution records in south-east Kyushu by examination of museum collections [file zookeys-725-079-s001.pdf]

Supplement 1. List of voucher specimens of *Chaenogobius annularis* for distribution records in south-east Kyushu by examination of museum collections. Lots of specimens ordered according to longitude. Number of KPM-NR indicates photographs of fresh specimens deposited in KPM.

Voucher number	Number of KPM-NR	Number of individuals	SL (mm)	Locality	Prefecture	Geographic coordinates	Collecting date
KAUM–I. 4800		1	24,5	Nagashima Town	Kagoshima	32°10'35"N, 130°10'56"E	16 July 2007
KAUM–I. 9104		1	39,3	Akune City	Kagoshima	31°56'50"N, 130°12'58"E	7 Apr. 2008
KAUM–I. 9106		1	43,4	Akune City	Kagoshima	31°56'50"N, 130°12'58"E	7 Apr. 2008
KAUM–I. 3686		1	24,8	Minami-kyushu City	Kagoshima	31°15'14"N, 130°26'40"E	14 May 2007
KAUM–I. 2946		1	31,8	Kagoshima City	Kagoshima	31°34'08"N, 130°34'04"E	20 Mar. 2007
KAUM–I. 2958		1	27,9	Kagoshima City	Kagoshima	31°34'08"N, 130°34'04"E	20 Mar. 2007
KAUM–I. 8130		9	26.8–33.4	Kagoshima City	Kagoshima	31°34'08"N, 130°34'04"E	24 Jan. 2008
KAUM–I. 9865		1	37,8	Kagoshima City	Kagoshima	31°34'08"N, 130°34'04"E	6 May 2008
KAUM–I. 10696		1	21,9	Kagoshima City	Kagoshima	31°36'15"N, 130°34'10"E	3 July 2008
KAUM–I. 11122		1	42,2	Yaku-shima island	Kagoshima	30°20'03"N, 130°39'50"E	9 Aug. 2008
KAUM–I. 3858		1	24,6	Chiringa-shima island	Kagoshima	31°16'37"N, 130°40'21"E	19 May 2007
KAUM–I. 5282		1	40,8	Tanega-shima island	Kagoshima	30°28'53"N, 130°52'52"E	1 Aug. 2007
KAUM–I. 76951		1	44,9	Tanega-shima island	Kagoshima	30°37'24"N, 130°56'56"E	19 Feb. 2015
KAUM–I. 68469		1	42,3	Tanega-shima island	Kagoshima	30°37'26"N, 130°56'57"E	25 Jan. 2015
KAUM–I. 88533		1	46,2	Tanega-shima island	Kagoshima	30°45'45"N, 131°04'28"E	5 June 2016
KAUM–I. 84358		1	14,3	Uchinoura Bay	Kagoshima	31°17'29"N, 131°06'59"E	29 Mar. 2016
KAUM–I. 84359		1	14,3	Uchinoura Bay	Kagoshima	31°17'29"N, 131°06'59"E	29 Mar. 2016
KAUM–I. 84360		1	14,9	Uchinoura Bay	Kagoshima	31°17'29"N, 131°06'59"E	29 Mar. 2016
KAUM–I. 84361		1	12,9	Uchinoura Bay	Kagoshima	31°17'29"N, 131°06'59"E	29 Mar. 2016
KAUM–I. 84362		1	12,3	Uchinoura Bay	Kagoshima	31°17'29"N, 131°06'59"E	29 Mar. 2016
KAUM–I. 11902		1	26,2	Shibushi Bay	Kagoshima	31°27'46"N, 131°09'31"E	20 July 2008
KAUM–I. 11905		1	28,3	Shibushi Bay	Kagoshima	31°27'46"N, 131°09'31"E	20 July 2008
KPM-NI 42853		1	35,3	Nichinan City	Miyazaki	31°40'59"N, 131°27'39"E	4 July 2016
KPM-NI 42854		2	28.5–29.7	Nichinan City	Miyazaki	31°40'59"N, 131°27'39"E	4 July 2016
KPM-NI 42855	179154	1	39,8	Miyazaki City	Miyazaki	31°48'11"N, 131°28'18"E	20 Aug. 2016
KPM-NI 42856		2	30.5–33.1	Miyazaki City	Miyazaki	31°48'11"N, 131°28'18"E	20 Aug. 2016
KPM-NI 42851		1	53,1	Miyazaki City	Miyazaki	31°48'13"N, 131°28'40"E	31 May 2016
KPM-NI 42852		2	32.2–33.5	Miyazaki City	Miyazaki	31°48'13"N, 131°28'40"E	31 May 2016
KPM-NI 42844	179147	1	52,9	Miyazaki City	Miyazaki	31°47'43"N, 131°28'40"E	10 Apr. 2016
KPM-NI 42845	179148	1	46,0	Miyazaki City	Miyazaki	31°47'43"N, 131°28'40"E	10 Apr. 2016
KAUM–I. 9601		1	49,8	Miyazaki City	Miyazaki	31°47'24"N, 131°28'48"E	26 Apr. 2008
KAUM–I. 9602		1	40,9	Miyazaki City	Miyazaki	31°47'24"N, 131°28'48"E	26 Apr. 2008
KAUM–I. 21427		1	41,7	Miyazaki City	Miyazaki	31°47'24"N, 131°28'48"E	17 July 2009
KAUM–I. 21442		1	37,9	Miyazaki City	Miyazaki	31°47'24"N, 131°28'48"E	17 July 2009
KAUM–I. 21443		1	35,9	Miyazaki City	Miyazaki	31°47'24"N, 131°28'48"E	17 July 2009
KAUM–I. 21444		1	39,3	Miyazaki City	Miyazaki	31°47'24"N, 131°28'48"E	17 July 2009
KAUM–I. 21447		1	29,7	Miyazaki City	Miyazaki	31°47'24"N, 131°28'48"E	17 July 2009
KAUM–I. 21448		1	33,8	Miyazaki City	Miyazaki	31°47'24"N, 131°28'48"E	17 July 2009
KAUM–I. 21449		1	22,7	Miyazaki City	Miyazaki	31°47'24"N, 131°28'48"E	17 July 2009
KAUM–I. 21450		1	22,8	Miyazaki City	Miyazaki	31°47'24"N, 131°28'48"E	17 July 2009
KAUM–I. 21458		1	22,3	Miyazaki City	Miyazaki	31°47'24"N, 131°28'48"E	17 July 2009
KAUM–I. 21460		1	36,7	Miyazaki City	Miyazaki	31°47'24"N, 131°28'48"E	17 July 2009
KAUM–I. 21461		1	32,4	Miyazaki City	Miyazaki	31°47'24"N, 131°28'48"E	17 July 2009
KAUM–I. 56680		1	28,5	Miyazaki City	Miyazaki	31°47'08"N, 131°29'20"E	6 Oct. 2013
KPM-NI 42841	179144	1	49,1	Kadogawa Bay	Miyazaki	31°28'15"N, 131°40'59"E	5 July 2015
KPM-NI 42843	179146	1	16,4	Kadogawa Bay	Miyazaki	31°28'17"N, 131°41'02"E	5 July 2015
KPM-NI 42850	179153	1	52,7	Nobeoka City	Miyazaki	31°30'54"N, 131°41'54"E	4 June 2016
KPM-NI 42857	179155	1	50,4	Nobeoka City	Miyazaki	31°30'54"N, 131°41'54"E	30 Aug. 2016
