# Supplementary material for: Southern limits of distribution of the intertidal gobies Chaenogobius annularis and C. gulosus support the existence of a biogeographic boundary in southern Japan (Teleostei, Perciformes, Gobiidae)
Source: Zookeys. 2017 Dec 29;(725):79–95. doi: 10.3897/zookeys.725.19952 (PMC5769740; doi:10.3897/zookeys.725.19952)
Supplement: Supplementary material 2 — List of voucher specimens of Chaenogobius gulosus for distribution records in south-east Kyushu by examination of museum collection [file zookeys-725-079-s002.pdf]

Supplement 2. List of voucher specimens of *Chaenogobius gulosus* for distribution records in south-east Kyushu by examination of museum collection. Lots of specimen ordered according to longitude. Number of KPM-NR indicating photographs of fresh specimen deposited in KPM.

Voucher number	Number of KPM-NR	Number of individuals	SL (mm)	Locality	Prefecture	Geographic coordinates	Collecting date
KAUM–I. 26410		1	19,6	Kamikoshiki-jima island	Kagoshima	31°51'42"N, 129°52'33"E	30 June 2008
KAUM–I. 26411		1	17,4	Kamikoshiki-jima island	Kagoshima	31°51'42"N, 129°52'33"E	30 June 2008
KAUM–I. 26412		1	20,5	Kamikoshiki-jima island	Kagoshima	31°51'42"N, 129°52'33"E	30 June 2008
KAUM–I. 26413		1	26,7	Kamikoshiki-jima island	Kagoshima	31°51'42"N, 129°52'33"E	30 June 2008
KAUM–I. 26414		1	24,6	Kamikoshiki-jima island	Kagoshima	31°51'42"N, 129°52'33"E	30 June 2008
KAUM–I. 300038		1	22,9	Minamisatsuma City	Kagoshima	31°25'04"N, 130°07'56"E	15 May 2017
KAUM–I. 4799		1	35,9	Nagashima Town	Kagoshima	32°10'35"N, 130°10'56"E	16 July 2007
KAUM–I. 8665		1	70,6	Minamisatsuma City	Kagoshima	31°24'37"N, 130°11'32"E	8 Mar. 2008
KAUM–I. 36198		1	54,4	Ichiki-kushikino City	Kagoshima	31°42'24"N, 130°15'44"E	13 June 2011
KAUM–I. 38702		1	56,2	Ichiki-kushikino City	Kagoshima	31°42'24"N, 130°15'44"E	13 June 2011
KAUM–I. 6847		1	65,8	Hirakawa, Kagoshima City	Kagoshima	-	21 Oct. 2007
KAUM–I. 61518		1	33,1	Kagoshima City	Kagoshima	31°33'N, 130°33'E	22 May 2014
KAUM–I. 61519		1	73,5	Kagoshima City	Kagoshima	31°33'N, 130°33'E	22 May 2014
KAUM–I. 20593		1	69,1	Kagoshima City	Kagoshima	31°33'51"N, 130°33'55"E	15 Oct. 2008
KAUM–I. 20594		1	83,7	Kagoshima City	Kagoshima	31°33'51"N, 130°33'55"E	15 Oct. 2008
KAUM–I. 20595		1	78,5	Kagoshima City	Kagoshima	31°33'51"N, 130°33'55"E	15 Oct. 2008
KAUM–I. 722		1	29,0	Kagoshima City	Kagoshima	31°34'08"N, 130°34'04"E	26 Sep. 2006
KAUM–I. 723		3	33.3–28.5	Kagoshima City	Kagoshima	31°34'08"N, 130°34'04"E	26 Sep. 2006
KAUM–I. 2613		7	6.2–10.2	Kagoshima City	Kagoshima	31°34'08"N, 130°34'04"E	18 Mar. 2007
KAUM–I. 2614		2	12.1–12.2	Kagoshima City	Kagoshima	31°34'08"N, 130°34'04"E	18 Mar. 2007
KAUM–I. 2947		1	58,5	Kagoshima City	Kagoshima	31°34'08"N, 130°34'04"E	20 Mar. 2007
KAUM–I. 2949		1	48,6	Kagoshima City	Kagoshima	31°34'08"N, 130°34'04"E	20 Mar. 2007
KAUM–I. 2952		1	45,5	Kagoshima City	Kagoshima	31°34'08"N, 130°34'04"E	20 Mar. 2007
KAUM–I. 2957		1	43,2	Kagoshima City	Kagoshima	31°34'08"N, 130°34'04"E	20 Mar. 2007
KAUM–I. 2959		1	42,3	Kagoshima City	Kagoshima	31°34'08"N, 130°34'04"E	20 Mar. 2007
KAUM–I. 2960		1	37,5	Kagoshima City	Kagoshima	31°34'08"N, 130°34'04"E	20 Mar. 2007
KAUM–I. 2961		1	43,2	Kagoshima City	Kagoshima	31°34'08"N, 130°34'04"E	20 Mar. 2007
KAUM–I. 2964		1	48,5	Kagoshima City	Kagoshima	31°34'08"N, 130°34'04"E	20 Mar. 2007
KAUM–I. 2983		1	82,8	Kagoshima City	Kagoshima	31°34'08"N, 130°34'04"E	20 Mar. 2006
KAUM–I. 4448		1	69,2	Kagoshima City	Kagoshima	31°34'08"N, 130°34'04"E	22 June 2007
KAUM–I. 8122		1	73,4	Kagoshima City	Kagoshima	31°34'08"N, 130°34'04"E	24 Jan. 2008
KAUM–I. 8123		1	61,4	Kagoshima City	Kagoshima	31°34'08"N, 130°34'04"E	24 Jan. 2008
KAUM–I. 8124		1	63,1	Kagoshima City	Kagoshima	31°34'08"N, 130°34'04"E	24 Jan. 2008
KAUM–I. 8125		1	61,5	Kagoshima City	Kagoshima	31°34'08"N, 130°34'04"E	24 Jan. 2008
KAUM–I. 8126		1	42,4	Kagoshima City	Kagoshima	31°34'08"N, 130°34'04"E	24 Jan. 2008
KAUM–I. 8127		1	40,5	Kagoshima City	Kagoshima	31°34'08"N, 130°34'04"E	24 Jan. 2008
KAUM–I. 8128		1	70,1	Kagoshima City	Kagoshima	31°34'08"N, 130°34'04"E	24 Jan. 2008
KAUM–I. 8129		1	53,4	Kagoshima City	Kagoshima	31°34'08"N, 130°34'04"E	24 Jan. 2008
KAUM–I. 9864		1	55,6	Kagoshima City	Kagoshima	31°34'08"N, 130°34'04"E	6 May 2008
KAUM–I. 46587		1	51,2	Kagoshima City	Kagoshima	31°34'08"N, 130°34'04"E	7 Apr. 2012
KAUM–I. 21000		1	76,9	Kagoshima City	Kagoshima	31°36'07"N, 130°34'12"E	9 May 2009
KAUM–I. 29016		1	69,6	Kagoshima City	Kagoshima	31°36'07"N, 130°34'12"E	29 Apr. 2010
KAUM–I. 29017		1	33,2	Kagoshima City	Kagoshima	31°36'07"N, 130°34'12"E	29 Apr. 2010
KAUM–I. 54586		1	35,1	Sakura-jima	Kagoshima	31°35'24"N, 130°35'31"E	26 May 2013
KAUM–I. 54587		1	32,8	Sakura-jima	Kagoshima	31°35'24"N, 130°35'31"E	26 May 2013
KAUM–I. 54588		1	37,0	Sakura-jima	Kagoshima	31°35'24"N, 130°35'31"E	26 May 2013
KAUM–I. 54589		1	32,7	Sakura-jima	Kagoshima	31°35'24"N, 130°35'31"E	26 May 2013
KAUM–I. 54590		1	35,1	Sakura-jima	Kagoshima	31°35'24"N, 130°35'31"E	26 May 2013
KAUM–I. 54591		1	34,2	Sakura-jima	Kagoshima	31°35'24"N, 130°35'31"E	26 May 2013
KAUM–I. 54592		1	34,0	Sakura-jima	Kagoshima	31°35'24"N, 130°35'31"E	26 May 2013
KAUM–I. 54593		1	30,2	Sakura-jima	Kagoshima	31°35'24"N, 130°35'31"E	26 May 2013

KAUM–I. 3026		1	46,5	Sakura-jima	Kagoshima	31°35'30"N, 130°35'39"E	21 Apr. 2007
KAUM–I. 3030		1	52,5	Sakura-jima	Kagoshima	31°35'30"N, 130°35'39"E	21 Apr. 2007
KAUM–I. 3031		1	29,8	Sakura-jima	Kagoshima	31°35'30"N, 130°35'39"E	21 Apr. 2007
KAUM–I. 3065		44	9.9–31.0	Sakura-jima	Kagoshima	31°35'30"N, 130°35'39"E	21 Apr. 2007
KAUM–I. 5000		1	30,9	Sakura-jima	Kagoshima	31°35'30"N, 130°35'39"E	24 July 2007
KAUM–I. 5001		1	27,6	Sakura-jima	Kagoshima	31°35'30"N, 130°35'39"E	24 July 2007
KAUM–I. 5002		1	29,9	Sakura-jima	Kagoshima	31°35'30"N, 130°35'39"E	24 July 2007
KAUM–I. 5003		1	27,4	Sakura-jima	Kagoshima	31°35'30"N, 130°35'39"E	24 July 2007
KAUM–I. 5004		1	33,4	Sakura-jima	Kagoshima	31°35'30"N, 130°35'39"E	24 July 2007
KAUM–I. 5005		1	33,7	Sakura-jima	Kagoshima	31°35'30"N, 130°35'39"E	24 July 2007
KAUM–I. 5006		1	29,9	Sakura-jima	Kagoshima	31°35'30"N, 130°35'39"E	24 July 2007
KAUM–I. 5007		1	29,6	Sakura-jima	Kagoshima	31°35'30"N, 130°35'39"E	24 July 2007
KAUM–I. 5008		1	29,2	Sakura-jima	Kagoshima	31°35'30"N, 130°35'39"E	24 July 2007
KAUM–I. 5009		1	34,0	Sakura-jima	Kagoshima	31°35'30"N, 130°35'39"E	24 July 2007
KAUM–I. 5010		1	37,0	Sakura-jima	Kagoshima	31°35'30"N, 130°35'39"E	24 July 2007
KAUM–I. 5011		1	28,8	Sakura-jima	Kagoshima	31°35'30"N, 130°35'39"E	24 July 2007
KAUM–I. 5597		1	36,0	Sakura-jima	Kagoshima	31°35'30"N, 130°35'39"E	30 July 2007
KAUM–I. 5598		1	35,9	Sakura-jima	Kagoshima	31°35'30"N, 130°35'39"E	30 July 2007
KAUM–I. 5599		1	36,4	Sakura-jima	Kagoshima	31°35'30"N, 130°35'39"E	30 July 2007
KAUM–I. 8967		1	59,1	Sakura-jima	Kagoshima	31°35'30"N, 130°35'39"E	27 Mar. 2008
KAUM–I. 8968		1	53,9	Sakura-jima	Kagoshima	31°35'30"N, 130°35'39"E	27 Mar. 2008
KAUM–I. 9375		1	53,0	Sakura-jima	Kagoshima	31°35'30"N, 130°35'39"E	19 Apr. 2008
KAUM–I. 9376		1	21,0	Sakura-jima	Kagoshima	31°35'30"N, 130°35'39"E	19 Apr. 2008
KAUM–I. 9876		1	21,2	Sakura-jima	Kagoshima	31°35'30"N, 130°35'39"E	19 Apr. 2008
KAUM–I. 88061		1	34,2	Sakura-jima	Kagoshima	31°35'35"N, 130°35'47"E	25 Aug. 1984
KAUM–I. 88062		1	38,0	Sakura-jima	Kagoshima	31°35'35"N, 130°35'47"E	21 Apr. 1989
KAUM–I. 88074		1	36,6	Sakura-jima	Kagoshima	31°35'35"N, 130°35'47"E	10 Dec. 1984
KAUM–I. 88076		1	41,3	Sakura-jima	Kagoshima	31°35'35"N, 130°35'47"E	10 Dec. 1984
KAUM–I. 15713		1	49,8	Sakura-jima	Kagoshima	-	19 Oct. 1960
KAUM–I. 15857		1	71,5	Sakura-jima	Kagoshima	-	19 Oct. 1960
KAUM–I. 15860		1	67,9	Sakura-jima	Kagoshima	-	19 Oct. 1960
KAUM–I. 15915		1	72,5	Sakura-jima	Kagoshima	-	19 Oct. 1960
KAUM–I. 47291		1	39,4	Chiringa-shima island	Kagoshima	31°16'37"N, 130°40'20"E	25 May 2012
KAUM–I. 3857		1	34,7	Chiringa-shima island	Kagoshima	31°16'37"N, 130°40'20"E	19 May 2007
KAUM–I. 62128		1	96,5	Tanega-shima island	Kagoshima	30°44'38"N, 130°59'46"E	13 June 2014
KAUM–I. 62129		1	82,4	Tanega-shima island	Kagoshima	30°44'38"N, 130°59'46"E	13 June 2014
KPM-NI 42846	179149	1	34,6	Nobeoka City	Miyazaki	32°30'47"N, 131°40'60"E	22 May 2016
KPM-NI 42842	179145	1	38,8	Kadogawa Bay	Miyazaki	32°28'17"N, 131°41'02"E	5 July 2015
KPM-NI 42847	179150	1	45,3	Nobeoka City	Miyazaki	32°30'28"N, 131°41'14"E	28 May 2016
KPM-NI 42848	179151	1	44,7	Nobeoka City	Miyazaki	32°30'28"N, 131°41'14"E	28 May 2016
KPM-NI 42849	179152	1	31,7	Nobeoka City	Miyazaki	32°30'28"N, 131°41'14"E	28 May 2016
KPM-NI 42858	179156	1	89,5	Nobeoka City	Miyazaki	32°30'59"N, 131°42'01"E	31 Aug. 2016
