# Supplementary material for: Southern limits of distribution of the intertidal gobies Chaenogobius annularis and C. gulosus support the existence of a biogeographic boundary in southern Japan (Teleostei, Perciformes, Gobiidae)
Source: Zookeys. 2017 Dec 29;(725):79–95. doi: 10.3897/zookeys.725.19952 (PMC5769740; doi:10.3897/zookeys.725.19952)
Supplement: Supplementary material 3 — List of voucher specimens of Chaenogobius annularis from quantitative surveys at rockpools on the eastern coasts of Kyushu [file zookeys-725-079-s003.pdf]

Supplement 3. List of voucher specimens of *Chaenogobius annularis* from quantitative surveys at rockpools on the eastern coasts of Kyushu. KPM-NI and KPM-NR indicates the number of the specimen and its photograph respectively.

KPM-NI	KPM-NR	Number of individuals	SL (mm)	Site	Season
42959		14	16.3–20.2	Oita	Spring
42960	179224	1	17,3	Oita	Spring
42963		5	16.4–20.1	Oita	Spring
42964	179226	1	17,6	Oita	Spring
42980		11	14.4–17.4	Oita	Spring
42981		26	15.9–18.0	Oita	Spring
42982		29	15.1–19.8	Oita	Spring
42983	179239	1	42,9	Oita	Spring
42984		9	15.9–20.3	Oita	Spring
42986		2	16.3–16.7	Oita	Spring
42987		11	14.8–18.4	Oita	Spring
43000		1	23,1	Oita	Autumn
43001		1	25,0	Oita	Autumn
43002		1	23,2	Oita	Autumn
43003		1	23,6	Oita	Autumn
43008		1	23,0	Oita	Autumn
43009		1	22,3	Oita	Autumn
43010		1	23,2	Oita	Autumn
43011		1	27,1	Oita	Autumn
43012		1	22,7	Oita	Autumn
43013		1	24,2	Oita	Autumn
43014		1	25,2	Oita	Autumn
43015		1	23,3	Oita	Autumn
43039		1	23,2	Oita	Autumn
43040		1	27,2	Oita	Autumn
43041		1	24,9	Oita	Autumn
43042		1	23,1	Oita	Autumn
43043		1	27,4	Oita	Autumn
43044		1	24,4	Oita	Autumn
43045		1	23,1	Oita	Autumn
43046		1	21,8	Oita	Autumn
43047		1	25,0	Oita	Autumn
43048		1	23,8	Oita	Autumn
43049		1	22,1	Oita	Autumn
43050		6	18.4–21.8	Oita	Autumn
43052		4	23.6–26.9	Oita	Autumn
43055		2	21.9–25.2	Oita	Autumn
42929		6	13.2–17.2	N-Miyazaki	Spring
42934		4	16.1–17.5	N-Miyazaki	Spring
42936		2	15.1–18.8	N-Miyazaki	Spring
42939	179211	1	38,9	N-Miyazaki	Spring
42942	179214	1	16,0	N-Miyazaki	Spring
42943	179215	1	14,4	N-Miyazaki	Spring
42944	179216	1	13,2	N-Miyazaki	Spring
42945	179217	1	13,2	N-Miyazaki	Spring
42948	179220	2	45.5–45.8	N-Miyazaki	Spring
42949		1	37,5	N-Miyazaki	Spring
42950		5	13.5–17.1	N-Miyazaki	Spring
42954		4	40.3–43.9	N-Miyazaki	Spring
42955		1	17,6	N-Miyazaki	Spring
43063		4	43.7–50.2	N-Miyazaki	Autumn

43081		1	39,8	N-Miyazaki	Autumn
43082		1	37,0	N-Miyazaki	Autumn
43083		1	37,0	N-Miyazaki	Autumn
43084		1	44,4	N-Miyazaki	Autumn
43085		1	33,8	N-Miyazaki	Autumn
43086		1	30,3	N-Miyazaki	Autumn
43087		1	34,0	N-Miyazaki	Autumn
43088		1	36,0	N-Miyazaki	Autumn
43089		1	31,7	N-Miyazaki	Autumn
43101		1	44,9	N-Miyazaki	Autumn
43102		1	28,6	N-Miyazaki	Autumn
43103		1	36,4	N-Miyazaki	Autumn
43116		6	44.1–47.9	N-Miyazaki	Autumn
43117		7	34.2–41.3	N-Miyazaki	Autumn
43118		6	33.7–41.9	N-Miyazaki	Autumn
43119		7	32.2–36.0	N-Miyazaki	Autumn
43120		9	25.6–36.3	N-Miyazaki	Autumn
42890		55	14.4–17.6	S-Miyazaki	Spring
42899	179192	1	18,0	S-Miyazaki	Spring
42900	179193	1	16,9	S-Miyazaki	Spring
42901	179194	1	16,7	S-Miyazaki	Spring
42905		151	14.0–18.0	S-Miyazaki	Spring
42914		1	45,0	S-Miyazaki	Spring
42917	179203	1	17,1	S-Miyazaki	Spring
42918	179204	1	25,2	S-Miyazaki	Spring
42919		3	16.5–19.0	S-Miyazaki	Spring
