# Supplementary material for: Southern limits of distribution of the intertidal gobies Chaenogobius annularis and C. gulosus support the existence of a biogeographic boundary in southern Japan (Teleostei, Perciformes, Gobiidae)
Source: Zookeys. 2017 Dec 29;(725):79–95. doi: 10.3897/zookeys.725.19952 (PMC5769740; doi:10.3897/zookeys.725.19952)
Supplement: Supplementary material 4 — List of voucher specimens of Chaenogobius gulosus from quantitative surveys at rockpools on the eastern coasts of Kyushu [file zookeys-725-079-s004.pdf]

Supplement 4. List of voucher specimens of *Chaenogobius gulosus* from quantitative surveys at rockpools on the eastern coasts of Kyushu. KPM-NI and KPM-NR indicates the number of the specimen and its photograph respectively.

KPM-NI	KPM-NR	Number of individuals	SL (mm)	Site	Season
42961	179225	1	31,4	Oita	Spring
42962		1	27,1	Oita	Spring
42972	179234	1	27,7	Oita	Spring
42973	179235	1	74,6	Oita	Spring
42979		3	25.7–29.6	Oita	Spring
42985		1	28,9	Oita	Spring
42988		1	43,1	Oita	Autumn
42989		1	43,1	Oita	Autumn
42990		1	34,1	Oita	Autumn
42991		1	32,4	Oita	Autumn
42992		1	32,7	Oita	Autumn
42993		1	38,6	Oita	Autumn
42994		1	41,2	Oita	Autumn
42995		1	44,6	Oita	Autumn
42996		1	46,5	Oita	Autumn
42997		1	50,0	Oita	Autumn
42998		1	45,9	Oita	Autumn
42999		1	61,1	Oita	Autumn
43006		1	43,1	Oita	Autumn
43007		1	32,5	Oita	Autumn
43017		1	35,4	Oita	Autumn
43018		1	40,8	Oita	Autumn
43019		1	49,3	Oita	Autumn
43020		1	42,0	Oita	Autumn
43021		1	35,1	Oita	Autumn
43022		1	49,1	Oita	Autumn
43023		1	34,5	Oita	Autumn
43024		1	29,6	Oita	Autumn
43051		2	38.3–46.0	Oita	Autumn
43053		1	76,1	Oita	Autumn
43054		1	77,0	Oita	Autumn
43058		1	36,8	Oita	Autumn
43059		1	94,7	Oita	Autumn
42951	179221	1	73,0	N-Miyazaki	Spring
